# Effect of the Infill Density on 3D-Printed Geometrically Graded Impact Attenuators

**DOI:** 10.3390/polym16223193

**Published:** 2024-11-17

**Authors:** Mateus Q. dos Reis, Ricardo J. C. Carbas, Eduardo A. S. Marques, Lucas F. M. da Silva

**Affiliations:** 1 Departamento de Engenharia Mecânica e Gestão Industrial, Faculdade de Engenharia, Universidade do Porto, 4200-465 Porto, Portugalemarques@fe.up.pt (E.A.S.M.); lucas@fe.up.pt (L.F.M.d.S.); 2 Instituto de Ciência e Inovação em Engenharia Mecânica e Engenharia Industrial (INEGI), 4200-465 Porto, Portugal

**Keywords:** three-dimensional printing, fused deposition modelling (FDM), impact attenuator, crash box, impact loading, PLA, PC

## Abstract

Three-dimensional printing is widely becoming prevalent in various industries, including the automotive sector. As this technology advances, critical structures subjected to impact loads may also be produced using additive manufacturing. A key parameter in this technique is the infill density of the printed geometry, which directly affects mechanical properties such as strength, stiffness, and ductility. Functionally graded layouts present themselves as one of the best techniques to design effective impact attenuators. The present work combines these techniques and parameters to evaluate the behaviour of geometrically graded impact attenuators produced through additive manufacturing, with different infill densities for polylactic acid (PLA) and polycarbonate (PC) materials. The results obtained show an increase in the mechanical strength for both materials and all the infill densities when compared to reference quasi-static results.

## 1. Introduction

Additive manufacturing, commonly known as 3D printing, is changing the automotive industry due to the unique advantages provided by this technique, such as increased design flexibility, better cost efficiency, and process agility. Unlike traditional manufacturing methods, which often involve the machining of larger pieces subtracting additive manufacturing builds objects layer by layer, allowing for the creation of complex geometries that were previously too complex and/or expensive to produce [1,2]. Additional benefits from this technique include the possibility of rapid prototyping, which accelerates the design process and reduces the time for new vehicles to be developed [3,4]. Engineers and designers can easily manufacture and test parts, rework the designs, and finalize components without the long production times associated with traditional manufacturing [4,5]. Moreover, additive manufacturing allows a higher level of customization and personalization of vehicles, allowing some components to be tailored to individual/personal customer preferences, from interior fittings to performance parts [6,7]. Furthermore, additive manufacturing contributes to weight reduction, which is extremely relevant for reducing fuel consumption and emissions [8,9]. Through the optimization of parts and designs and utilizing lightweight materials, manufacturers can develop and produce stronger and lighter components, increasing the vehicle’s performance [10,11]. Additionally, 3D printing enables a more sustainable manufacturing process, once it can minimize material waste since only the necessary amount of material is used to build each part [12,13]. This efficiency not only reduces costs but also minimizes the environmental impact of the production process [14,15].

The infill density is a critical parameter in 3D printing that significantly affects the mechanical properties, amount of material used, and quality of the final printed object. Infill density refers to the amount of internal structure within a 3D-printed part, typically represented as a percentage. A higher infill density means more material is used inside the object, resulting in a denser and stronger print, whereas a lower infill density results in a lighter, less material-intensive object with varying degrees of strength and flexibility [16,17]. The choice of infill density directly impacts several aspects of 3D-printed objects, including their weight, strength, and production time. For applications requiring high strength and durability, such as functional prototypes or mechanical parts, a higher infill density is often preferred [18,19]. However, for non-structural models or parts where weight reduction is crucial, a lower infill density may be used [20,21]. Different infill patterns, such as honeycomb, grid, and triangular, can be used to optimize the strength-to-weight ratio of printed parts. These patterns not only influence the mechanical properties but also affect the printing speed and material consumption. The selection of an appropriate infill pattern and density is essential for balancing the trade-offs between material usage, structural integrity, and printing efficiency [22,23]. Finally, optimizing infill density can lead to significant cost savings in material and production time, making it an essential factor for economic efficiency in 3D printing [24,25]. By adjusting the infill settings, high-quality parts can be tailored to specific needs without material waste [1,2].

Several studies have been performed on the mechanical behaviour of 3D-printed materials, such as polylactic acid (PLA) and polycarbonate (PC), regarding how loading rate, impact direction, and geometric variations affect the materials’ behaviour. PLA is known for being easy to print, and possesses a relatively high tensile strength for a polymer, making it suitable for lightweight applications, while PC presents higher impact resistance, making it more suitable for applications that require larger toughness [26,27,28,29,30,31,32,33].

The mechanical properties of materials obtained by additive manufacturing are very sensitive to the strain rate, due to their polymeric nature. PLA tensile strength and modulus increase with higher strain rates due to the material’s brittleness. At lower testing speeds, PLA can present more plastic deformation before failure. Moreover, PLA brittleness limits the effectiveness under impact conditions. Polycarbonate is tougher and tends to perform well under a large range of loading rates and presents a stable ductile behaviour across a large range of loading speeds, avoiding the onset of early fracture. Higher test speeds may improve its impact resistance, but the behaviour of PC is less brittle in comparison to PLA [26,27,30,31,32,33].

Infill density is a major factor for the mechanical properties presented by polymeric materials. High infill densities lead to enhanced tensile and stiffness properties of PC and PLA under quasi-static conditions, due to the reinforcement effect caused by the larger amount of material. Under impact conditions, the tensile properties of PLA also increase, but the effect on strength is reduced, due to the material brittleness. However, for PC, higher infill densities promote increased tensile properties and mechanical resistance under quasi-static and impact conditions, according to the literature [34,35].

The direction of the impact, or more specifically for 3D-printed materials, the angle between the load and the printing direction, plays a relevant role in the materials’ response. PLA tends to be less resistant in the perpendicular direction (Z-axis) of the printed layer. This behaviour occurs due to the poor inter-layer bonding which causes delamination during the loading. However, PC exhibits a higher toughness and better interlayer adhesion, being less sensitive to the impact direction, even if some anisotropy is still present as for most the materials obtained by additive manufacturing, PLA and PC present higher strength when loaded in the printing direction [29,36].

The geometry of the specimens also influences the mechanical response, especially in complex structures and/or designs with thin walls. Parts with higher infill density and/or denser internal patterns usually exhibit higher mechanical strength. At the same time, thicker walls typically lead to higher mechanical resistance, especially for PLA which is more brittle and more susceptible to be sensitive to geometric variations. Moreover, complex geometries and sharp corners can act as stress concentrations, leading to early failure of the PLA [28,37,38].

The mechanical properties of PLA and PC obtained by additive manufacturing can vary depending on the printing orientation and/or relative loading direction. For the PLA, the tensile strength values decrease when varying from 0° to 90°. PLA usually presents tensile strength in the range of 45–60 MPa for 0°, and the value decreases to 30–45 MPa for 45° and even lower for 90°, due to the weaker interlayer bonding. Regarding the elongation at break, since PLA is a brittle material, the values are often around 2–8% at 0° and may be reduced for other printing orientations, according to the anisotropy of the printed layers. PLA exhibits a higher stiffness at 0°, and the Young’s modulus value is often 1.5–3.0 GPa, being reduced for other printing orientations. PC is a tougher material, and the average tensile strength is around 60–80 MPa at 0° and may decrease to 40–65 MPa for the printing orientations of 45° and 90°, due to interlayer bonding differences. PC presents higher elongation than PLA, with values around 10–15% for 0° orientation that may drop to 5–10% for the other printing orientations, according to the specific material composition and printing parameters. The average modulus of PC is usually around 1.8–2.5 GPa for 0° and is slightly lower for other printing orientations [30,34,39,40].

Impact attenuators, essential components in automotive safety systems, have traditionally been manufactured using conventional methods such as moulding and machining. However, additive manufacturing has emerged as a transformative technology capable of optimizing the designs, production, and performance of impact attenuators. Unlike traditional manufacturing techniques, which involve complex tooling and limited design flexibility, 3D printing allows for the creation of complex geometries with customized tailored internal structures that optimize energy absorption during impacts [1,16]. This work presents the experimental results of geometrically graded impact attenuators obtained by additive manufacturing tested under quasi-static and impact conditions. The infill density was variated in order to evaluate the effect on the behaviour and failure load of the specimens under different conditions. The novelty of the present work is the evaluation of FDM specimens that combine graded tapering and hollowing in the geometry of a crash box, with a complete novel optimized design. Although additive manufacturing has been extensively studied, the application of additive manufacturing for impact attenuators is a new field of research, with many paths to be researched and explored. 

## 2. Materials and Methods

### 2.1. Materials

Two different materials were studied in this work, polylactic acid (PLA) and polycarbonate (PC). These materials are well-suited for additive manufacturing applications and provide quite distinct mechanical properties which have been characterized by the authors in a previous work [38]. The summary of the anisotropic properties of both materials is presented in Table 1.

### 2.2. Manufacturing Details 

The raw polymers were supplied in a filament form, and the specimens were manufactured by 3D printing, through the process of fused filament fabrication (FFF), also known as fused deposition modelling (FDM). The specimens were manufactured using an Original Prusa i3 MK3S+ 3D printer (Prague, Czech Repubic).

### 2.3. Specimens Details

**Figure 1:**
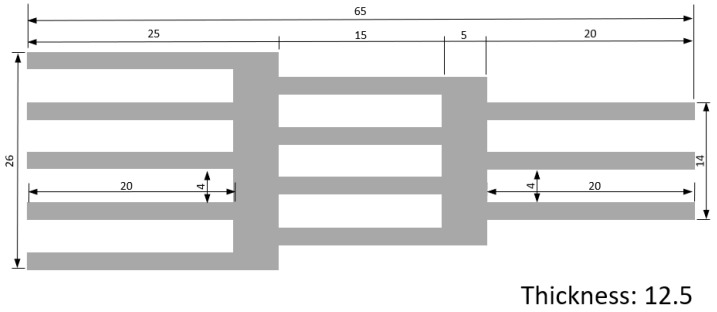
Impact attenuator layout (dimensions in mm).

The layout of the studied specimens is presented in Figure 1. This geometry is functionally graded through graded tapering (step sections) and graded hollowing, both of which can be used for increased energy absorption [41]. The stiffness and compliance also vary in every section, due to the difference in the number of plates. This geometry results in an impact attenuator shape, also known as crash boxes. These specimens are suitable to be tested under quasi-static and impact conditions.

**Figure 2:**
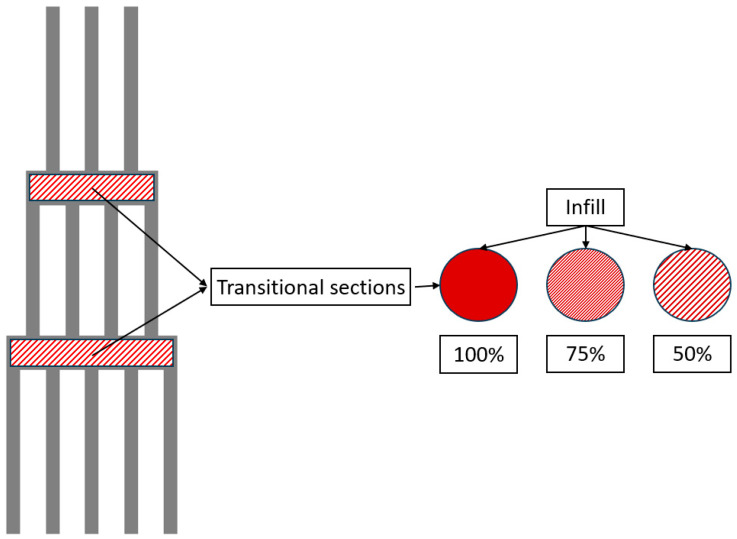
Variation in infill density details.

The specimens were manufactured with three different infill density conditions, 100%, 75%, and, 50% for the transitional sections, with a grid infill shape with a printing orientation of ±45°, to evaluate the effect of the different densities on the load response, as presented in Figure 2.

**Figure 3:**
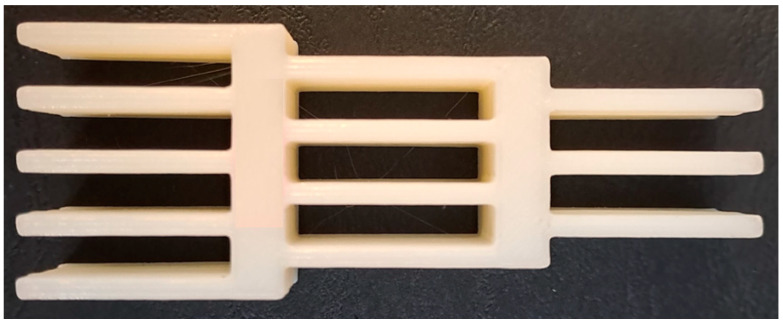
Impact attenuator after manufacturing.

The specimen obtained after manufacturing is presented in Figure 3. Moreover, the average mass for the impact attenuators with two different printed materials and three variable infill densities is presented in Table 2.

### 2.4. Quasi-Static Tests

For performing the compression quasi-static tests, a Shimadzu UH-X (Kyoto, Japan), a computer-controlled hydraulic servo system machine with a maximum load of 600 kN was used. The quasi-static tests were carried out at a constant crosshead rate of 1.0 mm/min.

### 2.5. Impact Tests

The drop-weight machine, which has been developed in-house by the authors specifically created for the study of adhesives and adhesive joints, was used to conduct the compression impact tests. It is capable of releasing a mass of up to 56 kg from a height of 1.27 m with a maximum test speed and load of 5 m/s and 650 kN, respectively [42]. To ensure complete joint failure, an impactor mass of 32.5 kg was employed throughout the tests, which were conducted at a speed of 3 m/s.

## 3. Experimental Results

### 3.1. Quasi-Static Results

**Figure 4:**
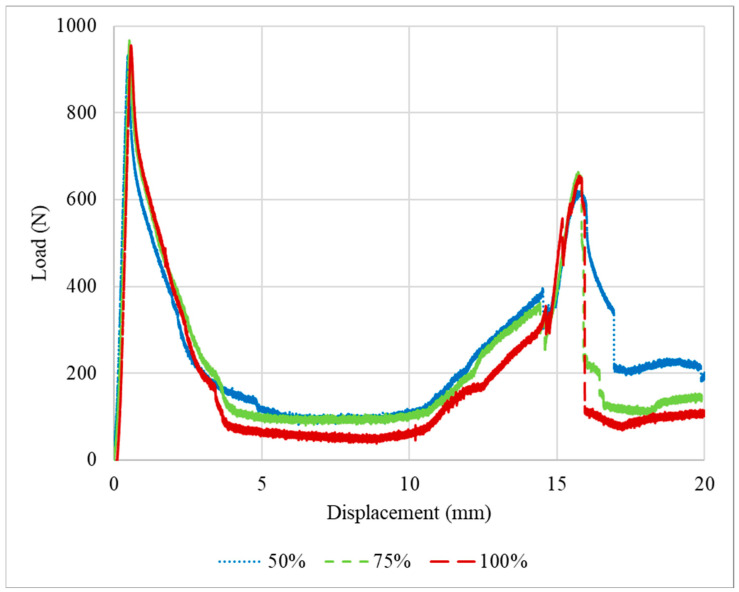
Representative load–displacement curves for the PLA impact attenuators under quasi-static conditions.

The P-δ curves obtained after the quasi-static tests performed for the PLA impact attenuators are presented in Figure 4. The tests were stopped after a displacement of 20 mm was attained. This limit was defined since performing compression testing indefinitely would not provide relevant additional results, resulting only in the compression of the middle section of the crash boxes until the thickness of the adherend plates was reached. 

**Figure 5:**
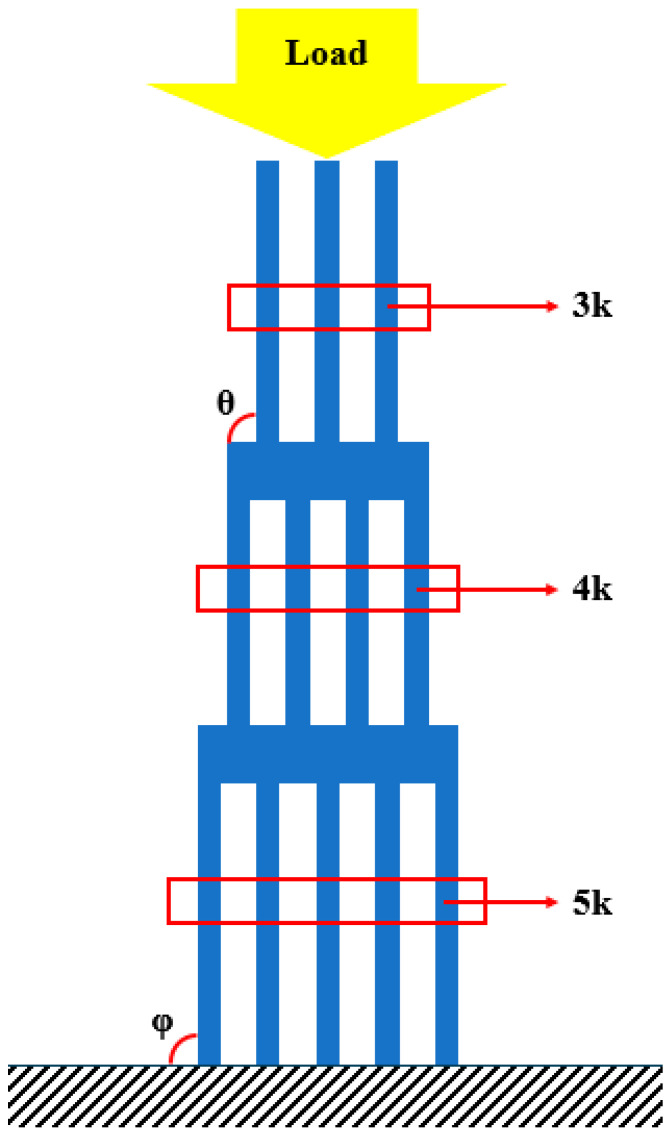
Sections stiffness and bending angles.

Specimens with 75% infill density presented a higher average failure load, less than 10% higher than the specimens with an infill density of 100% and 50%, respectively. Regarding the two peaks presented in the load-displacement curves, this occurs due to the asymmetrical compression of the impact attenuators. The first section has three thin plates, the middle Section 4, and the last Section 5. The middle section is restrained by the external sections which possess an additional degree of freedom on the free faces. Considering that each thin plate has a stiffness *k*, the first section has a stiffness of 3k, and consequently, a compliance of 1/3k, while the last section, also called the base, has a stiffness of *5k* and a compliance of 1/5k. During the loading scenario, in the first moment, the whole structure is under compression and some flexion/bending/buckling starts to happen in the top and base sections, until the elastic limit is reached, and due to the nature of the material, the load reduces. However, this is not the main reason for the major decrease in the load; this happened due to the plastic flexion/bending/buckling. With the increase in the bending angle *θ*, the load necessary to keep the deformation occurring reduces, due to the increase in the lever length, until the maximum bending angle possible for the geometry is obtained, which explains the notable reduction in the load after the first peak. Furthermore, as explained previously, the stiffness of the first section is lower than the last section, and the middle section is restrained. Thus, the first section exhibits a larger deformation at the beginning of the test in comparison with the stiffer base. After the initial deformation of the whole structure and geometrical accommodation of the load and displacements, both the top and bottom are deformed. However, at the top, the deformation keeps occurring at a higher rate, while at the base it occurs slowly until reaching a stable position. Only after the maximum bending angle for the top is reached will the bottom bending angle *φ* start deforming again, as presented in Figure 5, thus increasing the load and culminating in the secondary peak presented in the load-displacement curves.

**Figure 6:**
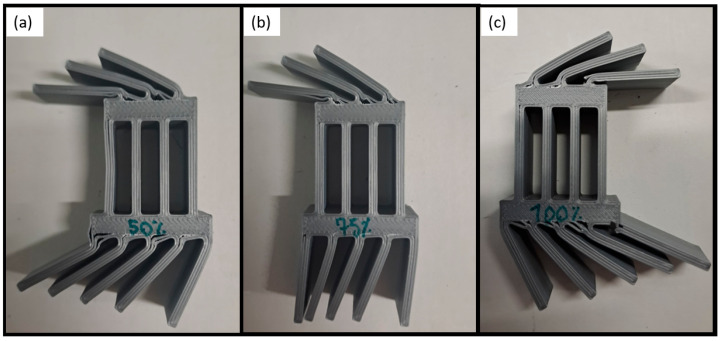
PLA impact attenuators with different infill densities after being tested under quasi-static conditions: (**a**) 50%, (**b**) 75%, and (**c**) 100%.

The specimens after being tested are presented in Figure 6. The difference in the behaviour of the specimens with different infill densities can be explained by the following. The transitional sections of the specimens with 50% infill have less material than the others. Hence, the strength of the section is lower, as is the failure load. For the 100% density, the section is too stiff and leads to early failure due to brittle behaviour. Under quasi-static conditions, according to the experimental results, PLA with 75% infill density seems to be the optimal solution regarding the higher failure load.

**Figure 7:**
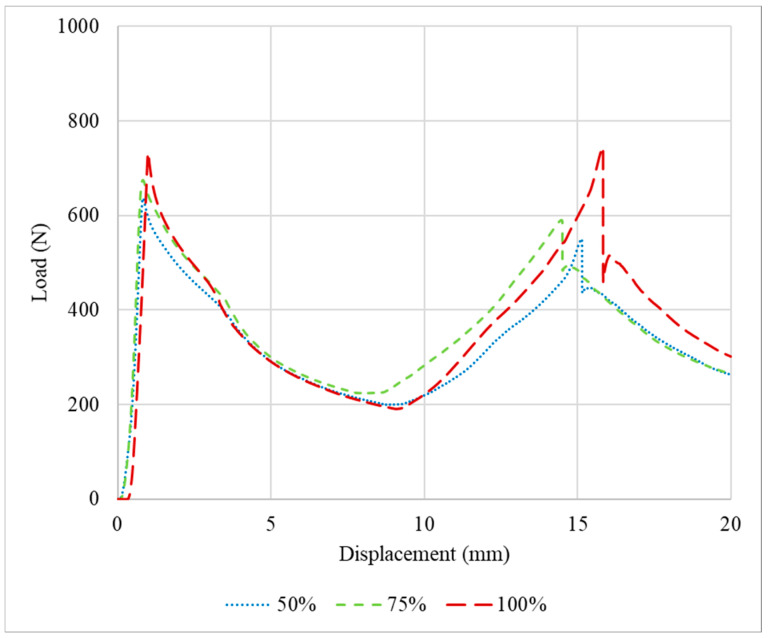
Load-displacement curves for the PC impact attenuators under quasi-static conditions.

**Figure 8:**
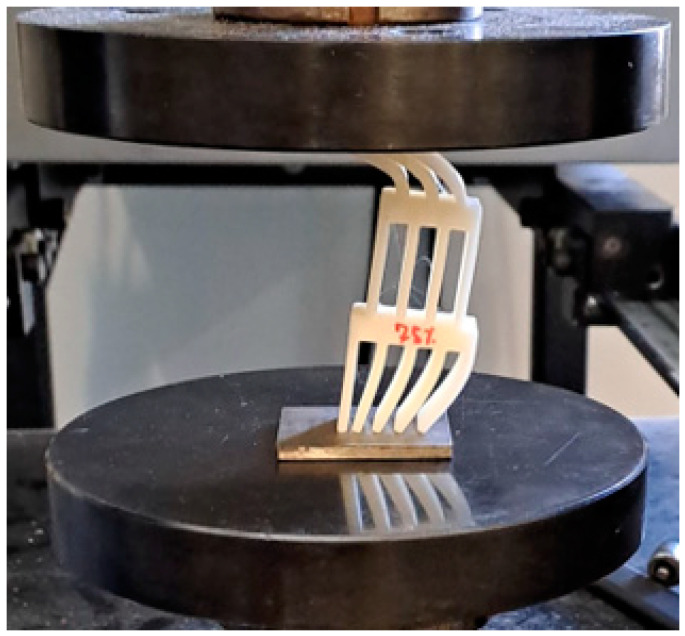
Asymmetrical bending during testing.

**Figure 9:**
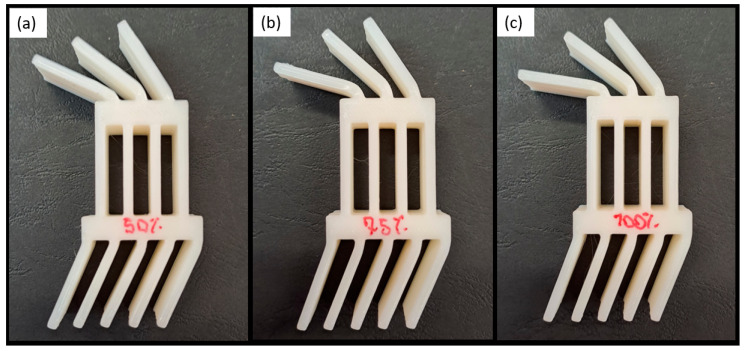
PC impact attenuators with different infill densities after being tested under quasi-static conditions: (**a**) 50%, (**b**) 75%, and (**c**) 100%.

**Figure 10:**
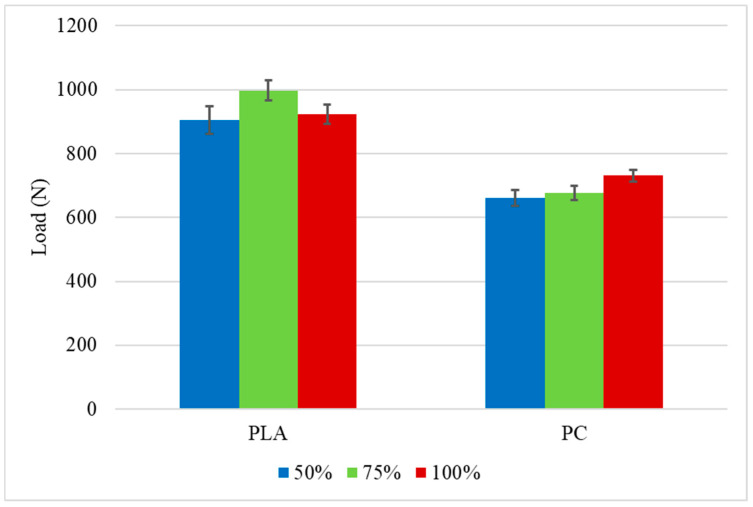
Average maximum load for the impact attenuators tested under quasi-static conditions.

The load-displacement curves for the PC specimens tested at 1 mm/min are presented in Figure 7. The same displacement limitation used for the PLA was applied. The higher failure load was obtained for the specimens with an infill density of 100%, approximately 8% higher than the crash boxes with a transitional section of 75% infill density and 10% higher than the specimens with 50% infill density, which presented statistically similar values to the 75%, considering the experimental deviation. The lower dispersion of the results for the different infill densities at the transitional area can be explained by the high toughness of PC, which makes them less susceptible to the variation in the infill density. Once again, two peaks are visible on the P-δ curves, which occurs for the same reason that was previously explained for PLA. The occurrence of this phenomenon during the test can be seen in Figure 8. The tested PC impact attenuators are presented in Figure 9. For this material, the higher failure load followed the trend of higher strength for the specimens with higher infill density; meanwhile, lower infill densities lead to lower strength. The summary of the average failure loads for both materials is presented in Figure 10. From the obtained results of the maximum loads of both materials, all the values obtained for the PLA were higher than the values obtained for the PC. This is aligned with the properties of the bulk materials obtained through the characterization of the polymers. Since the impact attenuators were not tested until the failure of the composite, the maximum load of PLA is higher than the PC because the elasticity modulus of the PLA is also higher.

### 3.2. Impact Results

**Figure 11:**
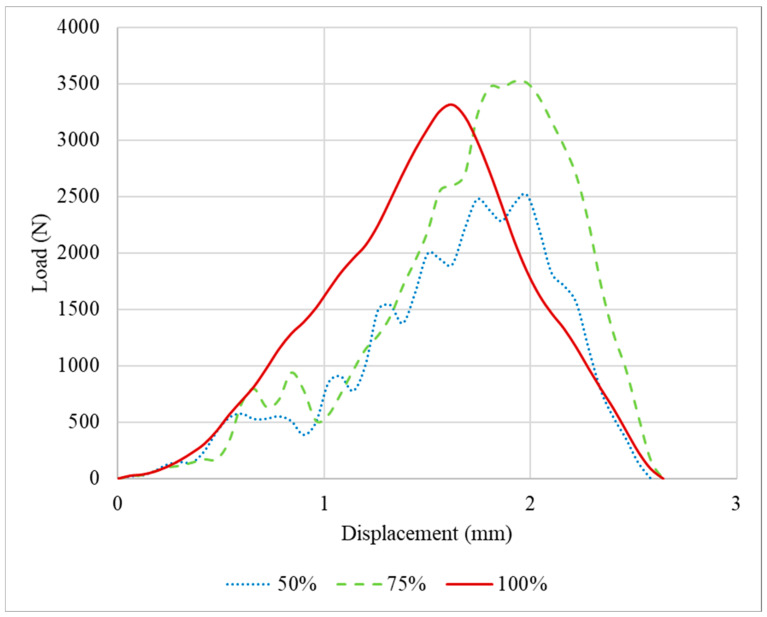
Load-displacement curves for the PLA specimens tested under impact conditions.

The load and displacement for the PLA tested under impact conditions are presented in Figure 11. As observed for the specimens tested under quasi-static conditions, the impact attenuators with 75% infill density presented the higher average failure load, 38% higher than the 50% infill density and 7% than the 100%. However, considering the deviation of the experimental data, there is an overlap between 75% and 100%. Regarding the displacement, for the three infill densities studied, the average value obtained was around 3 mm. 

**Figure 12:**
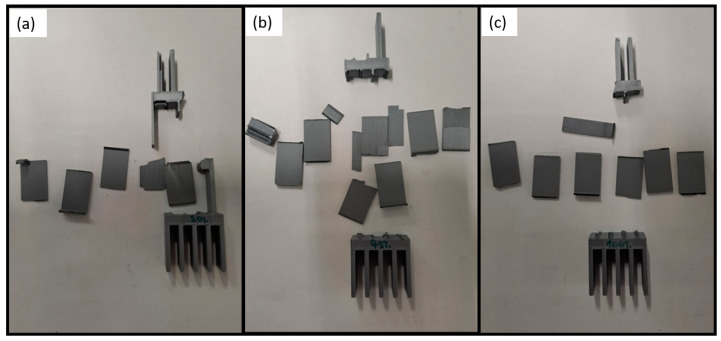
PLA impact attenuators with different infill densities after being tested under impact conditions: (**a**) 50%, (**b**) 75%, and (**c**) 100%.

Images of the specimens after testing are presented in Figure 12. As can be seen, no plastic deformation occurred, and all the fractures of the thin plates and transitional section were brittle. Once again, the difference in the average failure loads can be explained as a result of the same reasons as the PLA under quasi-static conditions. The specimens with an infill density of 50% have a lesser amount of material, leading to a lower structure strength. For the crash boxes with 100% infill, the specimens are too stiff, leading to a premature failure. The addition of the voids for the PLA with 75% infill provides an additional ductility to the transitional zones, increasing the ultimate strength, from avoiding the early fracture due to high embrittlement. Moreover, it should be highlighted that the base section remained intact for all the infill densities evaluated, highlighting the structural stability of this construction with PLA material.

**Figure 13:**
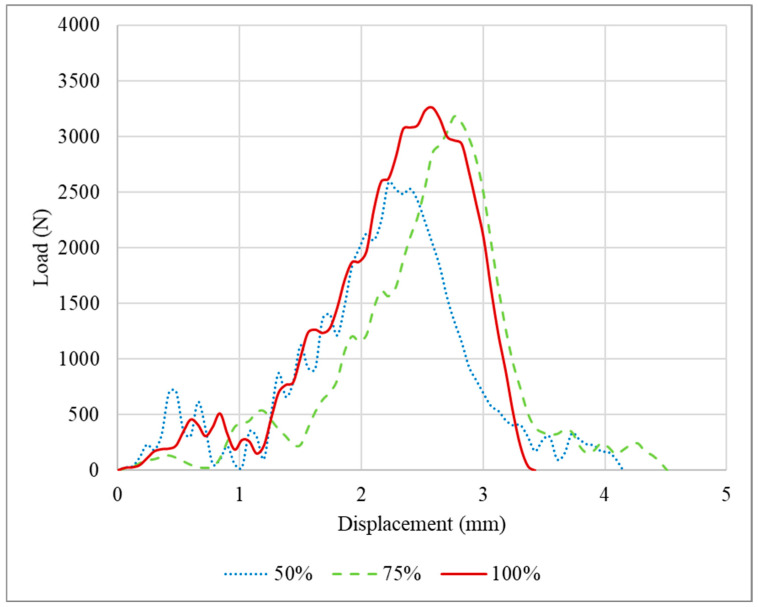
Load-displacement curves for the PC specimens tested under impact conditions.

The P-δ curves for the PC impact attenuators tested at 3 m/s are presented in Figure 13. The general trend for the quasi-static results was also observed for the specimens tested under impact conditions, with the average failure load higher for the pieces with higher infill density. 

**Figure 14:**
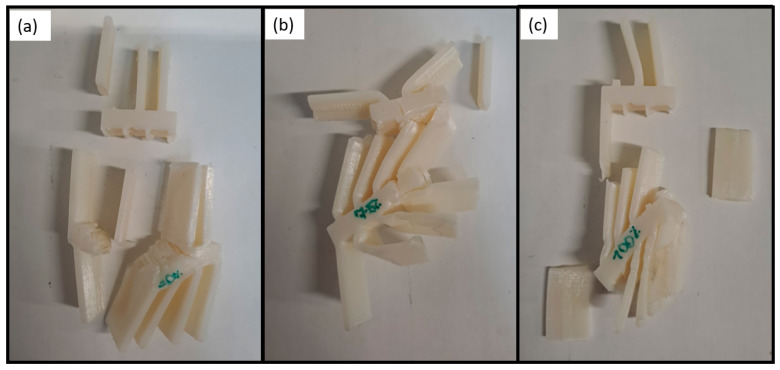
PC impact attenuators with different infill densities after being tested under impact conditions: (**a**) 50%, (**b**) 75%, and (**c**) 100%.

The PC impact attenuators with 100% infill presented average failure loads that were 7% and 38% higher than the crash boxes with 75% and 50% density, respectively. Once again, a small overlap in the experimental data deviation is presented for the 75% and 100% groups. Regarding the displacement, the 75% infill density presented higher average displacement values, which were 9% higher than the groups with the infill of 50% and 27% higher than the average values obtained for the specimens with an infill density of 100%. The reason for this behaviour is that a lower infill density leads to more ductility and/or plasticity of the PC. For the 50% group, this number of voids is too big and leads to a major decrease in the strength at the cost of a higher displacement, while for the 75% infill density, a good balance between bulk strength and ductility is obtained, leading to higher displacement. Figure 14 presents the PC impact attenuators after being impact tested. As can be seen, all groups suffered plastic deformation, which was also especially higher for the 75% infill specimens, due to the failure mechanism already explained in the displacement discussion. Furthermore, it should be addressed that for the PC, the whole structure is deformed and there is no integrity remaining, even at the base, for all the infill densities evaluated.

**Figure 15:**
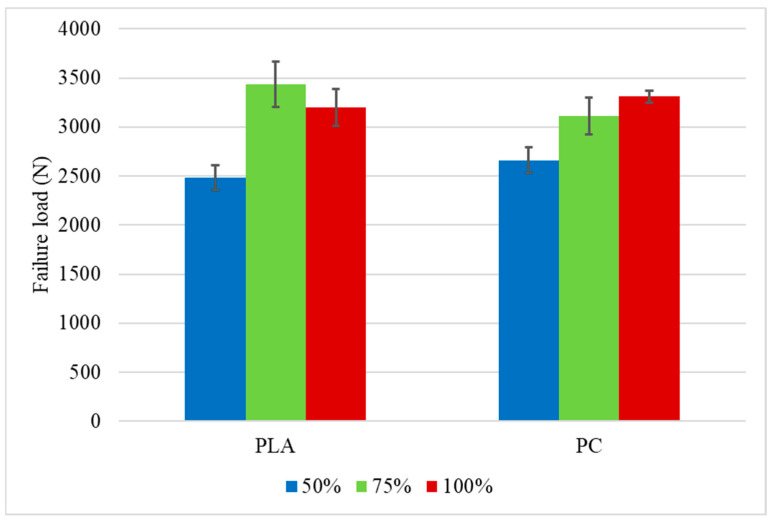
Average failure load for the impact attenuators tested under impact conditions.

**Figure 16:**
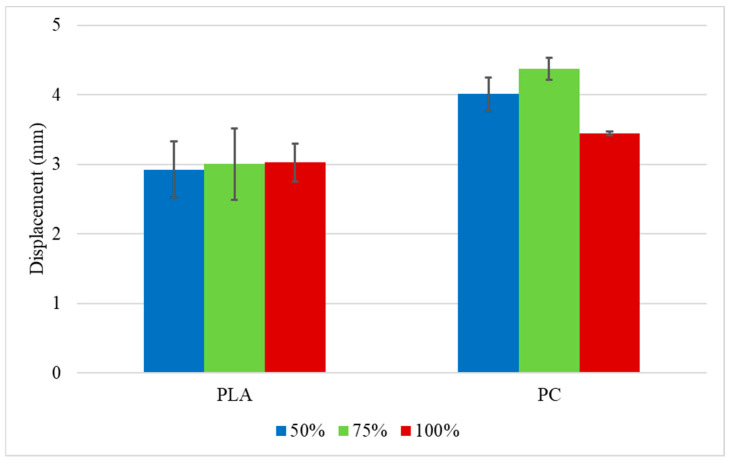
Average displacement for the impact attenuators tested under impact conditions.

The summary of the average failure loads and displacements obtained for both materials tested at impact are presented in Figure 15 and Figure 16, respectively. It can be seen that the average failure load for the PLA with an infill density of 75% was higher than the PC with the same density. For the other groups, the average failure load of the PC was higher. A higher displacement was also observed for all PC groups, which was already expected from the bulk material characterization since PC is more ductile than the PLA.

## 4. Discussion 

**Figure 17:**
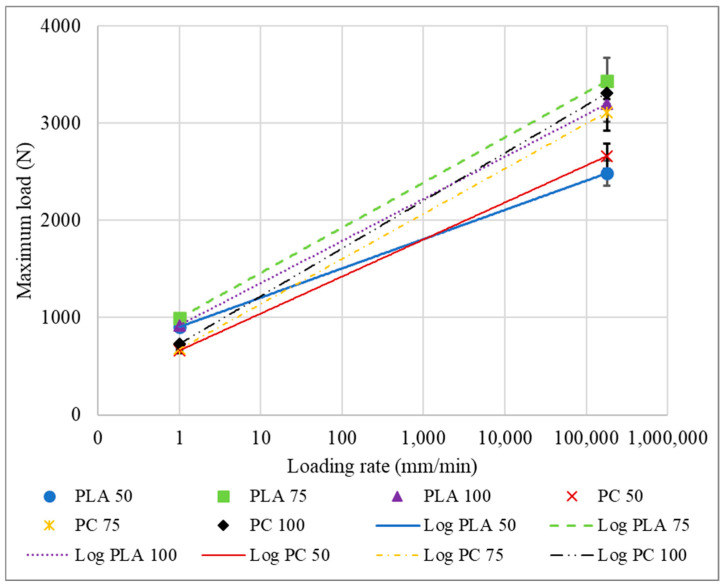
Maximum loads in function of the loading rate for the tested impact attenuators.

It is also important to compare the specimens in what concerns test speed, displacement rate, loading rate, and strain rate since dissimilar materials can present different behaviour and trends at different test conditions. Figure 17 shows the comparisons of the maximum loads. For the PLA, the infill rates of 75% and 100% presented a very similar level of sensitivity to the strain rate variation, with an increase in load values around 245% and 247%, respectively. Meanwhile, specimens with density in the transitional zone of 50% presented an increase of 175%. To estimate the values for other test speeds, log trends were applied to the test points. This approach to determining characteristics and numerical projection is accurate and widely recognized in the literature [43,44].

As observed for the PLA, the strain rate sensitivity for the PC with infill densities of 100% and 75% is quite similar, 353% and 360%, respectively. But unlike the PLA, for the PC, the 75% infill was the most sensitive. For the PC of 50%, the increase in the load between quasi-static and impact conditions was 302%. It can also be seen that for overall infill densities, the PC is more sensitive to the displacement rate than the PLA. Moreover, the ratio between the different infill groups of PLA presented a higher value in comparison to the PC.

## 5. Conclusions

Additive manufacturing is increasingly being used by a variety of industries, such as the automotive industry. It is expected that with the increase in this technology application, critical structures submitted to impact load may be also manufactured by 3D printing. For this technique, one of the main design parameters is the infill density of the printed geometry, which is directly correlated to the mechanical properties, strength, stiffness, and ductility of the produced parts. Hence, the adjustment of those parameters is critical for the energy absorption and weight of the structures. Moreover, from the literature, functionally graded layouts can be used for the development and production of a suitable design of impact attenuators. As can be seen from all the above information, the purpose of the present paper was to combine all of those techniques and parameters together and evaluate the behaviour of geometrically graded impact attenuators obtained by additive manufacturing with different infill densities for a transitional zone between the section with different stiffness and compliances. From the experimental results obtained, the following can be concluded:

1. The experimental results for the PLA specimens tested at quasi-static conditions demonstrate that an infill density of 75% has the highest average load—10% higher than the groups with 100% and 50% infill density. This is mainly due to the trade-off between strength and ductility. The PC specimens tested at quasi-static conditions presented a higher maximum load at a 100% infill density, closely followed by 75% and 50%, respectively. PC was shown to be less sensitive to the variation in the infill destiny at the transitional sections, due to the higher toughness of the material.

2. Concerning the results under impact conditions for the PLA specimens, the same trend was observed for the quasi-static results, with the 75% group presenting the highest average failure loads, 38% higher than the 50% infill density and 7% higher than the 100%, but with some statistical overlap between 75% and 100%. Regarding the displacement, all three groups presented an average displacement of 3mm, due to the material’s brittle behaviour under impact conditions for all the groups. The impact attenuators manufactured with PC presented higher average failure loads for the 100% infill specimen, with loads 7% and 38% higher than the crash boxes with 75% and 50% density, respectively. The same trend was observed for quasi-static conditions. The displacement was higher for the 75% infill density, 9% higher than the group with an infill of 50%, and 27% higher than the average values obtained for the specimens with an infill density of 100%.

3. For the PLA, comparing the maximum loads for quasi-static and impact conditions, it was observed an increase in all values under impact, 175%, 245%, and 247% were observed for the infill densities of 50%, 75%, and 100%, respectively. A similar increase in the higher loading rates was observed for the PC, with increases of 302%, 360%, and 353% for the infill densities of 50%, 75%, and 100%, respectively.

## Figures and Tables

**Table 1:** Anisotropic properties of the polymers (adipate from [36]).

**PLA**			
**Printing Orientation (°)**	**Tensile strength (MPa)**	**Elongation (%)**	**Young’s modulus (MPa)**
0	49.0	4.8	1920
45	37.0	5.7	1727
90	18.4	1.8	1022
**PC**			
**Printing Orientation (°)**	**Tensile strength (MPa)**	**Elongation (%)**	**Young’s modulus (MPa)**
0	72.0	9.0	1665
45	66.0	10.5	1650
90	65.5	8.2	1634

**Table 2:** Average mass for the different printed materials and infill densities evaluated.

Material	PLA	PC
Infill Density (%)	Mass (g)	
50	9.38	9.23
75	9.8	9.60
100	10.18	10.02

## Data Availability

To obtain the data presented in this work, please contact the corresponding author.
